# Feasibility of a 2-Part Substance Use Screener Self-Administered by Patients on Paper: Observational Study

**DOI:** 10.2196/52801

**Published:** 2024-06-25

**Authors:** Joanna Kramer, Timothy E Wilens, Vinod Rao, Richard Villa, Amy M Yule

**Affiliations:** 1 Department of Psychiatry Boston Medical Center Boston, MA United States; 2 Department of Psychiatry Massachusetts General Hospital Boston, MA United States

**Keywords:** patient reported outcome measures, patient reported outcomes, substance use screening, paper and pencil screening, screening, tobacco, prescription medication, medication, substance use, care, mental health, symptoms

## Abstract

**Background:**

Measurement-based care in behavioral health uses patient-reported outcome measures (PROMs) to screen for mental health symptoms and substance use and to assess symptom change over time. While PROMs are increasingly being integrated into electronic health record systems and administered electronically, paper-based PROMs continue to be used. It is unclear if it is feasible to administer a PROM on paper when the PROM was initially developed for electronic administration.

**Objective:**

This study aimed to examine the feasibility of patient self-administration of a 2-part substance use screener—the Tobacco, Alcohol, Prescription medications, and other Substances (TAPS)—on paper. This screener was originally developed for electronic administration. It begins with a limited number of questions and branches to either skip or reflex to additional questions based on an individual’s responses. In this study, the TAPS was adapted for paper use due to barriers to electronic administration within an urgent care behavioral health clinic at an urban health safety net hospital.

**Methods:**

From August 2021 to March 2022, research staff collected deidentified paper TAPS responses and tracked TAPS completion rates and adherence to questionnaire instructions. A retrospective chart review was subsequently conducted to obtain demographic information for the patients who presented to the clinic between August 2021 and March 2022. Since the initial information collected from TAPS responses was deidentified, demographic information was not linked to the individual TAPS screeners that were tracked by research staff.

**Results:**

A total of 507 new patients were seen in the clinic with a mean age of 38.7 (SD 16.6) years. In all, 258 (50.9%) patients were male. They were predominantly Black (n=212, 41.8%), White (n=152, 30%), and non-Hispanic or non-Latino (n=403, 79.5%). Most of the patients were publicly insured (n=411, 81.1%). Among these 507 patients, 313 (61.7%) completed the TAPS screener. Of these 313 patients, 76 (24.3%) adhered to the instructions and 237 (75.7%) did not follow the instructions correctly. Of the 237 respondents who did not follow the instructions correctly, 166 (70%) answered more questions and 71 (30%) answered fewer questions than required in TAPS part 2. Among the 237 patients who did not adhere to questionnaire instructions, 44 (18.6%) responded in a way that contradicted their response in part 1 of the screener and ultimately affected their overall TAPS score.

**Conclusions:**

It was challenging for patients to adhere to questionnaire instructions when completing a substance use screener on paper that was originally developed for electronic use. When selecting PROMs for measurement-based care, it is important to consider the structure of the questionnaire and how the PROM will be administered to determine if additional support for PROM self-administration needs to be implemented.

## Introduction

Measurement-based care (MBC) is a practice in clinical care that uses patient-reported outcome measures (PROMs) to identify individuals at risk for a disorder, quantify symptoms, and monitor symptoms over time [1]. PROMs can be self-administered and completed by patients on paper or electronically on a device or web-based platform [2]. Although paper-based PROMs are quite accessible, namely, because they do not require modifications to the electronic health record (EHR) to facilitate electronic administration [3], internet access, or technological literacy [2], there has been a shift within health care toward electronic administration of PROMs [3]. The shift toward electronic self-administration has followed the large-scale adoption of EHRs [4] since these systems can identify patients who are due to complete PROMs, streamline administration, improve ease of access to patient-reported outcome (PRO) responses for clinical decision-making, and monitor quality at the clinic and health care system levels [5,6]. Indeed, the integration of PROM into the EHR addresses important implementation barriers to MBC in clinic practice since PRO responses are immediately available to clinicians during the visit, results are easily interpreted within systems that automatically score responses, and results can be monitored within the EHR over time [6].

As electronic administration of PROMs became more common, new substance use screening questionnaires were specifically developed and validated for electronic administration [7-10]. To minimize the burden associated with completing a PROM, these questionnaires begin with a limited number of questions and skip or reflex to additional questions based on an individual’s initial responses [7-10]. One of these 2-part questionnaires—the Tobacco, Alcohol, Prescription medications, and other Substance (TAPS) screener—was also successfully integrated into the EHR and implemented in the primary care setting with electronic self-administration on tablets [11].

While electronic administration of PROMs that is automatically integrated with the EHR may ease patient and staff burdens associated with MBC, this system of screening may not be accessible in all health care settings. This type of system requires an information technology team that can help build and maintain the electronic PRO system, which can take a significant amount of time and expense and can also lead to recurring technological challenges once implemented [2,12,13]. In health safety net hospitals or federally qualified health centers, there may be fewer resources to incorporate the necessary information technology changes needed to systematically screen patients using electronic devices integrated with the EHR [14]. With this in mind, the most feasible screening option in these settings may be to continue the self-administration of PROMs on paper.

However, very little is known about the feasibility of administering a PROM on paper that was specifically developed to be administered electronically. We, therefore, aimed to examine questionnaire completion rates and adherence to questionnaire instructions when new patients completed the 2-part substance use TAPS screener on paper in an outpatient behavioral health clinic within a health safety net hospital. The clinic chose to use the TAPS screener because they were evaluating individuals at high risk for a co-occurring substance use disorder and needed a PROM that broadly assessed for multiple substances within 1 questionnaire to inform the initial treatment plan and referrals.

## Methods

### Overview

New patients aged 18 years and older presenting to an urgent care behavioral health clinic within an urban, public safety net hospital between August 2021 and March 2022 were given a paper packet by the front desk staff when they checked in for their appointment. This referral-based clinic provided urgent behavioral health services to adults experiencing a mental health crisis or needing medication to treat a psychiatric disorder or substance use disorder. The paper packet is part of standard care in this clinic and contains a 1-page intake form and 3 PROMs screeners assessing depression (the Patient Health Questionnaire-9) [15], anxiety (the Generalized Anxiety Disorder-7) [16], and substance use (TAPS) [7] to complete prior to their appointment. PROMs were administered on paper because there were no resources in this setting to administer PROMs electronically and immediately provide clinicians with the questionnaire results.

Research on survey design and presentation of questions on paper was used to derive the paper version of the TAPS from the web-based version [17] (see also Multimedia Appendix 1). The TAPS has 2 parts: part 1 of the TAPS assesses tobacco use, alcohol use, prescription medication misuse, and illicit substance use in the past 12 months. When use of a substance is endorsed, up to 4 additional yes or no questions are asked in part 2 for each substance endorsed to assess for problems associated with use in the past 3 months (up to 27 questions in total). In part 2, a yes response is scored a 1 and a no response is scored a 0. A score for each substance assessed in part 2 is calculated with corresponding categories of no risk (total score 0), problem use (total score 1), or higher risk (total score ≥2). To mimic the electronic questionnaire branching logic that either skips or reflexes to additional questions based on responses in part 1, written instructions were provided that indicated which questions respondents should answer in part 2 based on their response to the questions in part 1.

The front desk staff instructed patients to complete the packet and to hand it to their clinician when they were called into their appointment. The staff provided these instructions in English and Spanish, and the packets were provided in both languages. The goal of having patients complete the packet prior to their appointment was for their clinician to review the PRO responses and use this information during the appointment to inform the initial treatment plan and referrals. Following the appointment, clinicians would return the packet to a designated shelf in the waiting room for the research staff to collect. The research staff reviewed returned packets and scored them if this had not already been done by a clinician. The research staff maintained a deidentified database that detailed the completion and error rates of those who had completed some, all, or none of the PROMs screeners. A screener was considered incomplete if 1 or more questions were skipped.

**Figure 1:**
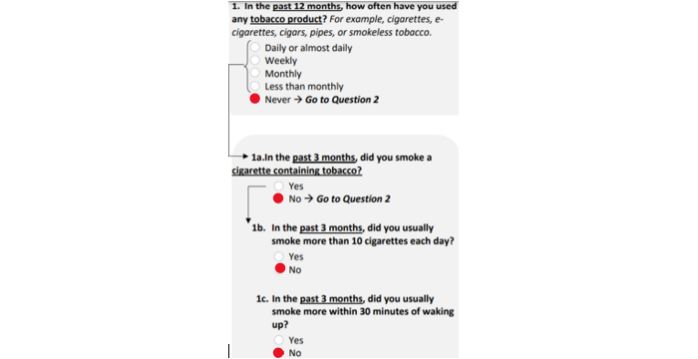
The questionnaire was not completed according to instructions because the respondent answered more questions than needed. However, their score was not impacted since they answered the TAPS part 2 questions (1a through 1c) in a consistent manner to TAPS part 1 (question 1). TAPS: Tobacco, Alcohol, Prescription medications, and other Substance.

**Figure 2:**
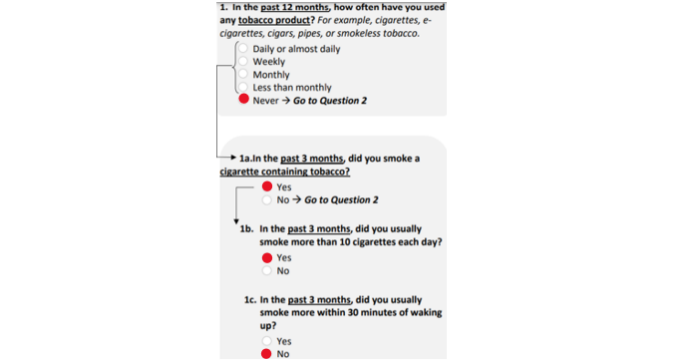
The questionnaire was not completed according to instructions because the respondent answered more questions than needed. Their score was impacted when they answered these additional questions since they answered the TAPS part 2 questions (1a through 1c) in an inconsistent manner compared to TAPS part 1 (question 1). TAPS: Tobacco, Alcohol, Prescription medications, and other Substance.

More detailed information was collected regarding completion patterns for the TAPS screener. This included whether individuals accurately completed the TAPS according to the instructions and whether or not their TAPS score was impacted based on how they responded. For example, if someone answered “never” to the use of a substance in part 1, no follow-up part 2 questions needed to be answered for that substance. However, when the TAPS was completed on paper, respondents saw all of the potential part 2 questions. Examples of completion patterns where the instructions were not followed and the score was not impacted (Figure 1), as well as when the score was impacted (Figure 2), are shown.

Since the deidentified database created to track PROMs completion and adherence to questionnaire instructions did not include patient demographic information, a retrospective EHR chart review was conducted to collect this information. Demographic information for all new patients who were seen in the clinic while the PROMs were tracked was extracted from the medical record and included age, race, ethnicity, sex, and the type of insurance. These demographic variables were classified according to how these items were categorized in the EHR.

### Ethical Considerations

The Boston University Medical Campus or Boston Medical Center institutional review board approved this research and deemed this study exempt and not requiring informed consent, as all information was deidentified and there was no direct interaction with human participants (H-43045).

## Results

A total of 507 new patients were seen in the clinic between August 2021 and March 2022. These patients had a mean age of 38.7 (SD 16.6) years, and 258 (50.9%) were male. They were predominantly Black (n=212, 41.8%), White (n=152, 30.0%), and non-Hispanic or non-Latino (n=403, 79.5%). Most of the patients were publicly insured (n=411, 81.1%; Table 1).

**Table 1:** Demographic characteristics of new patients seen in an urgent care behavioral health clinic between August 2021 and March 2022, when observational data were collected on the completion patterns for the 2-part TAPS^a^ questionnaire when self-administered on paper to patients (N=507).

Demographic characteristics		Values
**Sex, n (%)**		
	Male	258 (50.9)
	Female	248 (48.9)
	Unknown	1 (0.2)
**Race, n (%)**		
	Asian	25 (4.9)
	Black or African American	212 (41.8)
	Native Hawaiian or Pacific Islander	1 (0.2)
	White	152 (30)
	Multiple	11 (2.2)
	Declined or not available	29 (5.7)
	Other (including Hispanic or Latino)	77 (15.2)
**Ethnicity, n (%)**		
	Not Hispanic or Latino	403 (79.5)
	Hispanic or Latino	96 (18.9)
	Unknown	8 (1.6)
Age (years), mean (SD)		38.7 (14.6)
**Insurance, n (%)**		
	Public	411 (81.1)
	Private	82 (16.2)
	Uninsured	5 (1)
	Unknown	9 (1.8)

^a^TAPS: Tobacco, Alcohol, Prescription medications, and other Substance.

**Figure 3:**
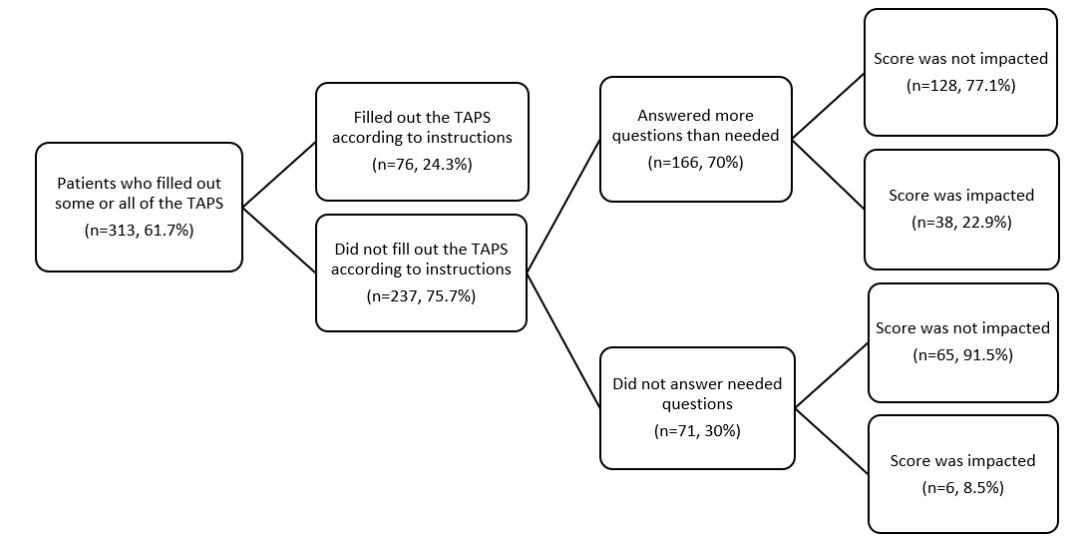
Observational data on the completion patterns for the 2-part TAPS questionnaire when self-administered on paper. TAPS: Tobacco, Alcohol, Prescription medications, and other Substance.

Out of the 507 new patients, 353 (69.6%) returned their paper packets, and 313 (61.7%) completed some or all of the TAPS screener. Among the 313 patients who completed some or all of the TAPS, 76 (24.3%) completed the full screener accurately according to the instructions on the paper form (Figure 3). Of the 237 individuals who did not complete the TAPS paper form according to the instructions, 166 (70.0%) answered more questions than required, and 71 (30.0%) did not answer required questions.

Out of the 166 individuals who answered more questions than required in TAPS part 2, most (n=128, 77.1%) did not overanswer in a way that affected their TAPS score (ie, responding “Never” to the part 1 question and then “Never” to the part 2 follow-up questions). However, 38 (22.9%) of those who answered more questions than needed responded to follow-up questions in TAPS part 2 in a way that contradicted their response in part 1 of the screener and ultimately affected their overall TAPS score.

Of the 71 patients who skipped needed questions on the paper TAPS form, 65 (91.5%) of these respondents did not have their TAPS score impacted by the questions they did respond to. For these individuals, the TAPS was not fully complete. However, for the questions they did answer, they answered more follow-up questions than needed in TAPS part 2, but not in a way that was contradictory to the questions they did respond to in TAPS part 1. There were 6 (8.5%) individuals who did not complete the entire TAPS, but whose score was impacted by the questions they did complete. Each of these individuals, for the questions they did answer, answered more follow-up questions than needed in TAPS part 2 and responded in a way that contradicted their part 1 TAPS response.

## Discussion

The goal of this study was to examine the feasibility of administering the 2-part TAPS questionnaire, originally created for electronic administration [7], on paper for patient self-administration. Many of the new patients who came to the outpatient behavioral health clinic for urgent evaluation completed the screener (313/507, 61.7%). The majority of these patients (237/313, 75.7%) did not adhere to the questionnaire instructions and answered more or less of the questions required. Furthermore, some of the patients who did not adhere to the questionnaire instructions (44/237, 18.6%) provided responses that contradicted their initial responses which subsequently affected their overall score and the corresponding category of level of risk associated with substance use.

Our finding that many patients in an outpatient behavioral health clinic completed the substance use PROM is similar to the existing literature on substance use screening in the behavioral health setting. A recent systematic review examined this topic and found that substance use screening rates in adult behavioral health clinics ranged from 48% to 100% [18]. Most of the studies in this review did not report on the method of screening administration, but the 1 study with paper administration reported screening 74.9% of patients [19]. Furthermore, our paper TAPS screening rate of 61.7% (313/507) is similar to the rate of electronic TAPS screening reported in primary care (n=67,042, 72%) [20].

Our finding that patients did not adhere to questionnaire instructions to either skip or answer additional questions is consistent with the broader literature that has shown errors of commission and omission is common when questionnaires that include skip instructions are self-administered on paper [21]. Similar to our findings, another study that reported on adherence to questionnaire instructions when individuals were asked questions about alcohol use, illicit drug use, and other sensitive health behaviors on paper, also found that it was more common for respondents to answer questions that they did not need to answer and less common for respondents to answer questions in a contradictory way [22].

Although it is unclear why patients in our sample did not follow the questionnaire instructions when self-administering a 2-part PROM on paper, other research suggests that health literacy may influence accurate completion of self-administered PROMs. Health literacy refers to one’s ability to use and comprehend information in a way that is beneficial to their health [23]. Those with lower health literacy may have a more difficult time understanding and communicating health-related needs [23]. For example, Al-Tayyib et al [22] found that individuals who scored the lowest on health literacy, when compared to those who scored highest, were 8 times more likely to answer questions about alcohol in a contradictory way when the questionnaire was self-administered on paper. Furthermore, Porter et al [24] observed that participants with low health literacy found it more burdensome to answer questions related to their health on paper when compared to participants with high health literacy. Since safety net hospitals typically serve marginalized or underserved populations [25], which generally experience lower health literacy rates than nonmarginalized populations [26], future work should collect information from patients in this setting while they complete the 2-part PROM to elicit feedback on the design and format of the paper questionnaire. This feedback will be important to guide strategies to support paper administration of a 2-part PROM that ensures accurate questionnaire completion and minimizes the burden to patients.

This study has a number of methodological limitations. Although research staff used a standardized form to track PROMs completion that allowed for free-text responses to describe patterns observed, they did not systematically categorize the types of errors made in TAPS screener completion until clear patterns began to be observed. Additionally, it is unknown why some individuals did not respond to the TAPS screener. Since demographic data for patients who completed the paper TAPS were not documented on the tracker, the study team is also missing potentially meaningful demographic trends in those who did and did not complete the paper TAPS. Further, it is evident from previous literature that health literacy plays a role in how individuals complete self-administered health screeners on paper, and literacy was not assessed in this study. This study was also conducted in 1 unique behavioral health urgent care setting and, therefore, might not be generalizable to other behavioral health settings or primary care.

Despite these limitations, our data support the feasibility of screening for substance use on paper in the outpatient behavioral health setting. Our data also highlight the challenge of poor adherence to questionnaire instructions when administering a 2-part substance use screener originally developed for electronic administration in a health safety net hospital setting. In the future, it is important to consider this challenge when adapting 2-part electronic screening questionnaires to paper and to implement strategies that minimize patient burden and ensure that accurate information is collected to inform an individual’s treatment plan. For 2-part screeners like the TAPS, it may be that part 2 needs to be completed with trained staff after a patient self-administers part 1.

## Appendix

Multimedia Appendix 1 Paper version of the two-part Tobacco, Alcohol, Prescription Medication, and Other Substance (TAPS) questionnaire that was used. Observational data were collected on the completion patterns for this questionnaire when self-administered on paper to patients in an outpatient behavioral health clinic within a health safety net hospital.

## Abbreviations

EHR: electronic health record
MBC: measurement-based care
PRO: patient-reported outcome
PROM: patient-reported outcome measure
TAPS: Tobacco, Alcohol, Prescription medications, and other Substance

## Data Availability

The data sets generated and analyzed during this study are available from the corresponding author on reasonable request.
