# Supplementary material for: Feasibility of a 2-Part Substance Use Screener Self-Administered by Patients on Paper: Observational Study
Source: JMIR Form Res. 2024 Jun 25;8:e52801. doi: 10.2196/52801 (PMC11234052; doi:10.2196/52801)
Supplement: Multimedia Appendix 1 [file formative_v8i1e52801_app1.pdf]

Supplement 1. Paper version of the two-part Tobacco, Alcohol, Prescription Medication, and Other Substance (TAPS) questionnaire that was used when observational data was collected on the completion patterns for this questionnaire when self-administered on paper to patients in an outpatient behavioral health clinic within a health safety net hospital

The Tobacco, Alcohol, Prescription medications, and other Substance Tool

This health survey will ask you questions about your use, if any, of tobacco, marijuana, alcohol, and other drugs. We ask this to all of our patients, because this information helps us provide you the best possible care. Please talk to your provider about your responses. Only your care team will have access to your responses unless there is a concern for your safety.

Instructions:

Mark your answers by filling in the circle next to the most accurate option.

Example: Question number 1

- ○ Option 1
- ● Option 2
- ○ Option 3

1. In the past 12 months, how often have you used any tobacco product? *For example, cigarettes, e-cigarettes, cigars, pipes, or smokeless tobacco.*

- ○ Daily or almost daily
- ○ Weekly
- ○ Monthly
- ○ Less than monthly
- ○ Never → ***Go to Question 2***

1a. In the past 3 months, did you smoke a cigarette containing tobacco?

- ○ Yes
- ○ No → ***Go to Question 2***

1b. In the past 3 months, did you usually smoke more than 10 cigarettes each day?

- ○ Yes
- ○ No

1c. In the past 3 months, did you usually smoke more within 30 minutes of waking up?

- ○ Yes
- ○ No

How many times did you check "yes" above (1a – 1c) ? ☐

2. In the past 12 months, how often have you had 4 or more drinks containing alcohol in one day? *One standard drink is about 1 small glass of wine (5 oz), 1 beer (12 oz), or 1 single shot of liquor.*

- ○ Daily or almost daily
- ○ Weekly
- ○ Monthly
- ○ Less than monthly
- ○ Never → ***Go to Question 3 on Page 2***

2a. In the past 3 months, did you have a drink containing alcohol? *One standard drink is about 1 small glass of wine (5 oz), 1 beer (12 oz), or 1 single shot of liquor.*

- ○ Yes
- ○ No → ***Go to Question 3 on Page 2***

2b. In the past 3 months, did you have 4

or more drinks containing alcohol in a day?

- ○ Yes
- ○ No

2c. In the past 3 months, have you tried and failed to control, cut down or stop drinking?

- ○ Yes
- ○ No

2d. In the past 3 months, has anyone expressed concern about your drinking?

- ○ Yes
- ○ No

How many times did you check "yes" above (2a-2d)? ☐

3. In the past 12 months, how often have you used any drugs including marijuana, cocaine or crack, heroin, methamphetamine (crystal meth), hallucinogens, ecstasy/MDMA?

- ◯ Daily or almost daily
- ◯ Weekly
- ◯ Monthly
- ◯ Less than monthly
- ◯ Never → *Go to Question 4 on page 3*

3a. In the past 3 months, did you use marijuana (hash, weed)?

- ◯ Yes
- ◯ No → *Go to Question 3d*

3b. In the past 3 months, have you had a strong desire or urge to use marijuana at least once a week or more often?

- ◯ Yes
- ◯ No

3c. In the past 3 months, has anyone expressed concern about your use of marijuana?

- ◯ Yes
- ◯ No

How many times did you check "yes" above (3a-3c)? ☐

3d. In the past 3 months, did you use cocaine, crack, or methamphetamine (crystal meth)?

- ◯ Yes
- ◯ No → *Go to Question 3g*

3e. In the past 3 months, did you use cocaine, crack, or methamphetamine (crystal meth) at least once a week or more often?

- ◯ Yes
- ◯ No

3f. In the past 3 months, has anyone expressed concern about your use of cocaine, crack, or methamphetamine (crystal meth)?

- ◯ Yes
- ◯ No

How many times did you check "yes" above (3d-3f) ? ☐

3g. In the past 3 months, did you use heroin?

- ◯ Yes
- ◯ No → **Go to Question 4 on Page 3**

3h. In the past 3 months, have you tried and failed to control, cut down or stop using heroin?

- ◯ Yes
- ◯ No

3i. In the past 3 months, has anyone expressed concern about your use of heroin?

- ◯ Yes
- ◯ No

How many times did you check "yes" above (3g-3i)? ☐

4. In the <u>past 12 months,</u> how often have you used any prescription medications just for the feeling, more than prescribed or that were not prescribed for you? *Prescription medications that may be used this way include: <u>Opiate pain relievers</u> (for example, OxyContin, Vicodin, Percocet, Methadone), <u>medications for anxiety or sleeping</u> (for example, Xanax, Ativan, Klonopin), <u>medications for ADHD</u> (for example, Adderall or Ritalin).*

- ◯ Daily or almost daily
- ◯ Weekly
- ◯ Monthly
- ◯ Less than monthly
- ◯ Never → ***Go to Question 5 on page 4***

4a. In the past 3 months, did you use a prescription opiate pain reliever (for example, Percocet, Vicodin) not as prescribed or that was not prescribed for you?

- ◯ Yes
- ◯ No → ***Go to Question 4d***

4b. In the <u>past 3 months</u>, have you tried and failed to control, cut down or stop using an opiate pain reliever?

- ◯ Yes
- ◯ No

4c. In the <u>past 3 months</u>, has anyone expressed concern about your use of an opiate pain reliever?

- ◯ Yes
- ◯ No

How many times did you check "yes" above (4a-4c)? ☐

4d. In the <u>past 3 months</u>, did you use a medication for anxiety or sleep (for example, Xanax, Ativan or Klonopin) not as prescribed or that was not prescribed for you?

- ◯ Yes → **Answer 4e and 4f**
- ◯ No → ***Go to Question 4g***

4e.In the <u>past 3 months</u>, have you had a strong desire or urge to use medications for anxiety or sleep at least once a week or more often?

- ◯ Yes
- ◯ No

4f. In the past <u>3 months</u>, has anyone expressed concern about your use of medication for anxiety or sleep?

- ◯ Yes
- ◯ No

How many times did you check "yes" above? ☐

4g. In the <u>past 3 months</u>, did you use a medication for ADHD (for example, Adderall, Ritalin) not as prescribed or that was not prescribed for you?

- ◯ Yes
- ◯ No → **Go to Question 5 on page 4**

4h.In the <u>past 3 months</u>, did you use a medication for ADHD (for example, Adderall, Ritalin) at least once a week or more often?

- ◯ Yes
- ◯ No

4i.In the <u>past 3 months</u>, has anyone expressed concern about your use of medication for ADHD (for example, Adderall or Ritalin)?

- ◯ Yes
- ◯ No

How many times did you check "yes" above (4g-4i)? ☐

If you did not answer "Never," to question 3 or question 4. answer the following question:

5. In the <u>past 3 months</u>, did you use any other illegal or recreational drug (for example, ecstasy/molly, GHB, poppers, LSD, mushrooms, special K, bath salts, synthetic marijuana ('spice'), whip-its, etc.)?

- ◯ Yes
- ◯ No

5a. In the <u>past 3 months</u>, what were the other drug(s) you used?
